# Detection of potentially novel paramyxovirus and coronavirus viral RNA in bats and rats in the Mekong Delta region of southern Viet Nam

**DOI:** 10.1111/zph.12362

**Published:** 2017-04-18

**Authors:** A. Berto, P. H. Anh, J. J. Carrique‐Mas, P. Simmonds, N. Van Cuong, N. T. Tue, N. Van Dung, M. E. Woolhouse, I. Smith, G. A. Marsh, J. E. Bryant, G. E. Thwaites, S. Baker, M. A. Rabaa, the VIZIONS consortium, Bach Tuan Kiet, Maciej F. Boni, Bui Duc Phu, James I Campbell, Dang Manh Hung, Dang Thao Huong, Dang Tram Oanh, Jeremy N. Day, Dinh Van Tan, H. Rogier van Doorn, Duong An Han, Jeremy J Farrar, Hau Thi Thu Trang, Ho Dang Trung Nghia, Hoang Bao Long, Hoang Van Duong, Huynh Thi Kim Thu, Lam Chi Cuong, Manh Hung, Thanh Phuong, Thi Phuc, Thi Phuong, Xuan Luat, Luu Thi Thu Ha, Ly Van Chuong, Mai Thi Phuoc Loan, Behzad Nadjm, Ngo Thanh Bao, Nguyen Canh Tu, Nguyen Dac Thuan, Nguyen Dong, Nguyen Khac Chuyen, Nguyen Ngoc An, Nguyen Ngoc Vinh, Nguyen Quoc Hung, Nguyen Thanh Dung, Nguyen Thanh Minh, Nguyen Thi Binh, Nguyen Thi Hong Tham, Nguyen Thi Hong Tien, Nguyen Thi Kim Chuc, Nguyen Thi Le Ngoc, Nguyen Thi Lien Ha, Nguyen Thi Nam Lien, Nguyen Thi Ngoc Diep, Nguyen Thi Nhung, Nguyen Thi Song Chau, Nguyen Thi Yen Chi, Nguyen Thieu Trinh, Nguyen Thu Van, Nguyen Van Hung, Nguyen Van Kinh, Nguyen Van Minh Hoang, Nguyen Van My, Nguyen Van Thang, Nguyen Van Thanh, Nguyen Van Vinh Chau, Nguyen Van Xang, Pham Ha My, Pham Thi Minh Khoa, Pham Thi Thanh Tam, Pham Van Lao, Pham Van Minh, Phan Van Be Bay, Motiur Rahman, Corinne Thompson, Ta Thi Dieu Ngan, Tran Do Hoang Nhu, Tran Hoang Minh Chau, Tran Khanh Toan, Tran My Phuc, Tran Thi Kim Hong, Tran Thi Ngoc Dung, Tran Thi Thanh Thanh, Tran Thi Thuy Minh, Tran Thua Nguyen, Tran Tinh Hien, Trinh Quang Tri, Vo Be Hien, Vo Nhut Tai, Vo Quoc Cuong, Voong Vinh Phat, Vu Thi Lan Huong, Vu Thi Ty Hang, Heiman Wertheim, Carlijn Bogaardt, Liam Brierley, Margo Chase‐Topping, Al Ivens, Lu Lu, Andrew Rambaut, Mark Woolhouse, Matthew Cotten, Bas B. Oude Munnink, Paul Kellam, My Vu Tra Phan, Lia van der Hoek, Martin Deijs, Maarten F. Jebbink, Seyed Mohammad Jazaeri Farsani, Karen Saylors, Nathan Wolfe

**Affiliations:** ^1^ Wellcome Trust Major Overseas Programme Oxford University Clinical Research Unit Ho Chi Minh City Viet Nam; ^2^ Centre for Tropical Medicine Nuffield Department of Clinical Medicine Oxford University Oxford UK; ^3^ Nuffield Department of Clinical Medicine University of Oxford Oxford UK; ^4^ Centre for Immunity, Infection & Evolution The University of Edinburgh Edinburgh UK; ^5^ Health and Biosecurity CSIRO, Australian Animal Health Laboratory Geelong Vic. Australia; ^6^ The London School of Hygiene and Tropical Medicine London UK

**Keywords:** bats, coronavirus, paramyxovirus, rats, Viet Nam, zoonotic viruses

## Abstract

Bats and rodents are being increasingly recognized as reservoirs of emerging zoonotic viruses. Various studies have investigated bat viruses in tropical regions, but to date there are no data regarding viruses with zoonotic potential that circulate in bat and rat populations in Viet Nam. To address this paucity of data, we sampled three bat farms and three wet markets trading in rat meat in the Mekong Delta region of southern Viet Nam. Faecal and urine samples were screened for the presence of RNA from paramyxoviruses, coronaviruses and filoviruses. Paramyxovirus RNA was detected in 4 of 248 (1%) and 11 of 222 (4.9%) bat faecal and urine samples, respectively. Coronavirus RNA was detected in 55 of 248 (22%) of bat faecal samples; filovirus RNA was not detected in any of the bat samples. Further, coronavirus RNA was detected in 12 of 270 (4.4%) of rat faecal samples; all samples tested negative for paramyxovirus. Phylogenetic analysis revealed that the bat paramyxoviruses and bat and rat coronaviruses were related to viruses circulating in bat and rodent populations globally, but showed no cross‐species mixing of viruses between bat and rat populations within Viet Nam. Our study shows that potentially novel variants of paramyxoviruses and coronaviruses commonly circulate in bat and rat populations in Viet Nam. Further characterization of the viruses and additional human and animal surveillance is required to evaluate the likelihood of viral spillover and to assess whether these viruses pose a risk to human health.

**Impacts**

- Bats and rodents are known reservoirs of highly diverse viral and bacterial populations, and a number of these viruses have been implicated in the emergence of novel infectious diseases in humans.
- The close proximity of humans to bat and rodent populations in South‐East Asia creates frequent opportunities for viral spillover and thus poses an unknown risk to human health.
- Viral surveillance in animal reservoirs is an important step to understanding the exposure of humans to potential zoonoses, the types of human–animal interaction that impact potential for spillover infection and the factors that determine the transmissibility and pathogenicity of viral zoonoses in humans.

## 1 Introduction

A large proportion of the agents of emergent infectious disease have a zoonotic origin. These zoonotic pathogens fall into a wide spectrum of genera, but due to their high genetic variability and wide circulation, RNA viruses arguably pose the most significant threat to human health. Further, due to the close proximity between animals and humans in low‐ and middle‐income countries, human populations in these locations are disproportionally at risk of exposure from these viral pathogens (Paterson et al., 2014). Although animal viruses with zoonotic potential have likely been circulating continually, a high number of zoonotic RNA viruses have been discovered in recent years. This phenomenon is not only dependent on more thorough and enhanced detection methods but is also likely associated with the changing behaviour of human populations and the closer proximity between humans and the animals that act as reservoirs for these viruses (Calisher, Childs, Field, Holmes, & Schountz, 2006; Moratelli & Calisher, 2015; Sasaki et al., 2012; Wong, Lau, Woo, & Yuen, 2007).

Multiple studies have implicated bats to be the most likely reservoir of numerous zoonotic viruses (Calisher et al., 2006; Moratelli & Calisher, 2015; Wong et al., 2007), and there are a range of contributing factors that may make so‐called “spillover” events, in which humans or other animals are infected, more probable. Many bat species, including the insectivorous *Scotophilus kuhlii* present across South and South‐East Asia (Bates et al., 2016), live in large groups with a range of social behaviours involving close and prolonged contact with others in the roost; this activity may facilitate the horizontal transmission of viruses between roost members. In addition, some bat species have a long life span (up to 35 years) and are able to travel long distances (e.g., *Eidolon helvum* can migrate >4,500 km), thus increasing the likelihood of exposure to infectious agents (Drexler, Corman, & Drosten, 2014; Escaffre, Borisevich, & Rockx, 2013; Plowright et al., 2015). The modification and destruction of natural habitats increase the likelihood of contact between bats and humans, thus providing new opportunities for interspecies viral exchange.

Bats are a known reservoir for rabies virus (Escobar et al., 2015; Jakava‐Viljanen et al., 2015; Moratelli & Calisher, 2015; Rocha, de Oliveira, Heinemann, & Gonçalves, 2015), and have recently been associated with other viral pathogens with a severe infection phenotype in humans. Notoriously, Nipah and Menangle viruses, both paramyxoviruses, have caused outbreaks in humans, horses and pigs in Australia, Cambodia, Malaysia, Bangladesh and India (Escaffre et al., 2013; Kulkarni, Tosh, Venkatesh, & Senthil, 2013). Furthermore, SARS coronavirus (SARS‐CoV) has been identified in Chinese bats, and it has been shown that the SARS‐CoV genome sequence generated from humans and civets during the 2002–2003 outbreak in China phylogenetically clustered within the bat associated group of SARS‐CoV‐like viruses (Calisher et al., 2006; Drexler et al., 2014; Moratelli & Calisher, 2015; Wong et al., 2007). Filoviruses, including Ebola and Marburg viruses, also pose a significant threat to human health, and despite human outbreaks being rare and sporadic, they are associated with a high case fatality rate (Hoffmann et al., 2013; Jayme et al., 2015; Kgaladi et al., 2013; Plowright et al., 2015).

Viet Nam is a middle‐income country in South‐East Asia. Bats are common in Viet Nam and may pose a threat from the circulation of zoonotic RNA viruses. Further, in parts of the country, rats are commonly trapped in the rice fields and sold live for consumption; 3,300–3,600 tonnes are sold nationally annually (Van Cuong et al., 2015). Rats and other rodents are also recognized as a reservoir of zoonotic viruses that can be transmitted via close contact with saliva, urine or faeces; the circulation of hantavirus has been reported in rats in Viet Nam (Van Cuong et al., 2015). As South‐East Asia is a hot spot for zoonotic viruses, the WT‐VIZIONS (Wellcome Trust‐Vietnamese Initiative on Zoonotic Infections) project (Rabaa et al., 2015) is aiming to generate data on the circulation of viral zoonotic pathogens that pose a risk to human health. With bats suspected to be the main reservoir for more than 200 viral species, 248 bat samples from three different guano farms in the south of Viet Nam (Dong Thap Province) were screened for paramyxoviruses, coronaviruses and filoviruses to assess the potential risk to human health. In addition, 270 rodent faecal samples from the same province were screened for paramyxoviruses and coronaviruses.

## 2 Materials and Methods

### 2.1 Bat samples

Insectivore bat colonies (*S. kuhlii*) rather than individual bats were targeted for screening; three bat guano farms were identified in three geographically distinct sampling locations in the south of Viet Nam. These farms were selected because they were the only bat guano farms identified and consenting to participate in regular sampling within the catchment area for Dong Thap Provincial Hospital, a primary site of ongoing human sampling for the identification of zoonotic infections under the VIZIONS project (Rabaa et al., 2015). The three sampling locations were visited every 12 weeks (one sampling event) on six occasions between 2012 and 2014. The farms were visited in the afternoon; to catch urine and faeces, a ten‐square metre section of plastic sheeting was placed under trees in which bats were roosting. The urine and faecal samples were collected from the plastic sheeting using a sterile Pasteur pipette for the urine and a plastic scoop for the faeces. These samples were then placed in a graduated 2‐ml tube (Sarstedt microtubes, Denmark) and transferred to the laboratory in Ho Chi Minh City for processing and/or storage at −80°C. At each sampling event, we aimed to collect 10–15 samples per farm. Depending on the roosting density and activity of the bats, we estimated the plastic sheeting could hold urine and faeces for up to 200 individual bats.

### 2.2 Rat samples

The rodent surveys and sampling were conducted as previously described by Van Cuong et al. (2015). Briefly, rats were purchased from three markets in Dong Thap Province in the Mekong Delta (two rat markets in Cao Lanh City and one market in Tam Nong District) every 4 months for 2 years, between 2012 and 2014. Specimens used in this investigation included faeces from 270 individual rats of four different rat species: *Bandicota indica* (*n* = 8), *Rattus argentiventer* (*n* = 234), *Rattus losea* (*n* = 20) *and Rattus tanezumi* (*n* = 8).

### 2.3 RNA extraction, PCR screening and sequencing

Total nucleic acid was extracted from the bat urine, bat faecal and rat faecal samples using an automated extracted system (MagNA Pure 96 System, Roche) with MagNA Pure 96 Viral RNA Small Volume Kits (Roche). Following extraction, nucleic acid samples were screened for target viruses using pan‐family primers for paramyxoviruses, coronaviruses and filoviruses. The primer sequences of viral targets and the nested PCR conditions were as previously described (Heaton et al., 1997; Ogawa et al., 2011; Poon & Peiris, 2008; Tong, Chern, Li, Pallansch, & Anderson, 2008). Positive controls were included for each run and provided by CSIRO Biosecurity Flagship & Australian Animal Health Laboratory, Geelong, Victoria, Australia. All PCR amplification‐positive samples were subsequently characterized by Sanger sequencing using an ABI 3700 sequencer in both forward and reverse directions using the primers from the second round nested PCR of each target virus.

### 2.4 Phylogenetic analysis

For both paramyxovirus and coronavirus, partial RdRp sequences were aligned with subsets of publicly available reference sequences, yielding an 84‐sequence data set for paramyxoviruses (344 bp; sites 14210–14553 within the genome), a 153‐sequence data set for alphacoronaviruses and a 93‐sequence data set for betacoronaviruses (407 bp; sites 15149–15565 within the genome), using Seqotron (Fourment & Holmes, 2016). Identical sequences and those with greater than 1% ambiguity or missing sequence data were subsequently removed from the alignments. jModelTest was run, and indicated the GTR+I+G model to be the best‐fit model of nucleotide substitution for all three data sets (Posada, 2009). Maximum‐likelihood phylogenies were run using RAxML under the GTRGAMMAI model with 1,000 bootstrap replications for each dataset (Stamatakis, 2006). Trees were visualized and annotated using FigTree (v1.4.2).

## 3 Results

### 3.1 Screening of bat samples for paramyxovirus, coronavirus and filovirus

Bat urine and faeces collected from three guano farms in the south of Viet Nam were extracted and subjected to PCR amplification for paramyxoviruses, coronaviruses and filoviruses. Four of 248 (1.6%) bat faecal samples and 11 of 222 (4.9%) urine samples tested positive for paramyxovirus RNA. The paramyxovirus‐positive faecal samples were identified in sampling round two on a single farm and in sampling round three on two farms. The positive urine samples were detected on three bat guano farms during the sixth round of sampling. Screening of bat faeces and urine for coronaviruses indicated that 55 of 248 (22.2%) of faecal samples were positive for coronavirus RNA; none of the urine samples contained detectable coronavirus RNA. Coronavirus RNA was detected in bat faecal samples isolated from all three sampled farms at all of the sampling time points. The bat urine and faecal samples were also screened for filoviruses, but no PCR amplification for filovirus RNA was detected despite amplification of appropriate controls. Specific information on coronavirus and paramyxovirus positivity by sampling site, time and species is indicated in Tables S1–S3.

### 3.2 Screening of rat samples for coronavirus and paramyxovirus

Of the 270 rat faecal samples screened for coronaviruses and paramyxoviruses, 12 of 270 (4.4%) were positive for coronavirus RNA, while none of the samples had detectable paramyxovirus RNA. The cDNA amplicons were sequenced and all twelve positive samples produced sequences suitable for phylogenetic analysis. Notably, all coronavirus‐positive faecal samples were obtained from rats of one species, *R. argentiventer*.

### 3.3 Phylogenetic relationships among Vietnamese coronaviruses and paramyxoviruses from bats and rats

**Figure 1:**
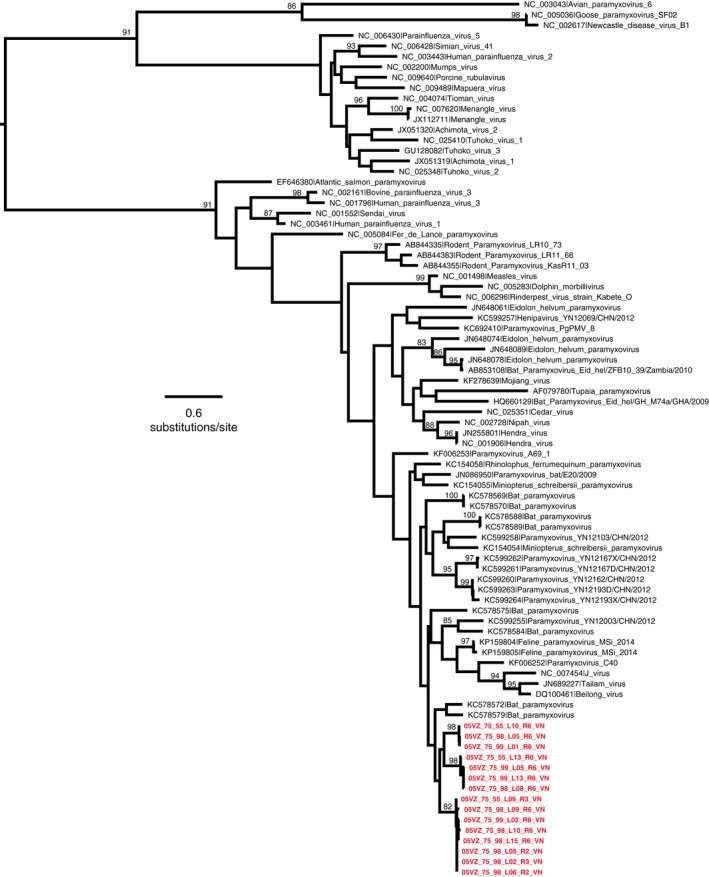
The phylogenetic relationships of paramyxoviruses sampled from Vietnamese bats. Maximum‐likelihood phylogeny constructed using RNA sequences from 15 Vietnamese bat paramyxovirus RNA‐positive samples and 69 reference sequences from the conserved region within the RNA‐dependent *RNA polymerase* (RdRp) gene (344 bp). The tree is mid‐point‐rooted for the purpose of clarity. Scale bar indicates the number of substitutions per site. Bootstrap support values are shown for nodes with ≥80% bootstrap support. Sequences from Vietnamese bats are indicated in red. [Colour figure can be viewed at http://wileyonlinelibrary.com]

Fifteen RNA sequences obtained from the bat paramyxovirus RNA‐positive samples (4/248 faecal samples and 11/222 urine samples) (GenBank accession numbers from KX092148 to KX092159; Table S4) were aligned with 69 reference sequences from the conserved region within the RNA‐dependent RNA polymerase (RdRp) gene (344 bp) accessed from GenBank. A maximum‐likelihood (ML) phylogeny suggested that all of these viruses were closely related and clustered with paramyxoviruses found in bat populations sampled globally, although low bootstrap values across much of the phylogeny indicate a largely unresolved evolutionary history of these viruses (Figure 1) using a short fragment of the RdRp gene alone. Based on ICTV classification, the bat paramyxoviruses determined in this study represented borderline novel species, with maximum nucleotide identities ranging from 77.8% to 81.97% compared to publicly available sequences (Table 1). All of our sequences showed the highest identity to recently described viruses isolated from various bat species in sub‐Saharan Africa.

**Table 1:** Maximum nucleotide and amino acid identities of Vietnamese bat paramyxovirus sequences relative to other known paramyxoviruses

Paramyxovirus	Highest nucleotide identity (%)	Closest match	Highest amino acid identity (%)	Closest match
05VZ_75_55_L13_R6_VN	80.18	KC578579|Kerivoula argentata|South Africa	92.11	KC578572|Eptesicus hottentotus|South Africa
05VZ_75_55_L09_R3_VN	78.91	KC578575|Nycteris thebaica|South Africa	93.75	KC578572|Eptesicus hottentotus|South Africa
05VZ_75_55_L10_R6_VN	80.47	KC578572|Eptesicus hottentotus|South Africa	92.94	KC578572|Eptesicus hottentotus|South Africa
05VZ_75_98_L02_R3_VN	79.26	KC578575|Nycteris thebaica|South Africa	93.86	KC578572|Eptesicus hottentotus|South Africa
05VZ_75_98_L05_R6_VN	80.78	KC578579|Kerivoula argentata|South Africa	93.14	KC578572|Eptesicus hottentotus|South Africa
05VZ_75_98_L06_R2_VN	79.26	KC578575|Nycteris thebaica|South Africa	93.86	KC578572|Eptesicus hottentotus|South Africa
05VZ_75_98_L08_R6_VN	80.82	KC578579|Kerivoula argentata|South Africa	92.45	KC578572|Eptesicus hottentotus|South Africa
05VZ_75_98_L09_R6_VN	77.33	KC578572|Eptesicus hottentotus|South Africa	91.23	KC578572|Eptesicus hottentotus|South Africa
05VZ_75_99_L13_R6_VN	80.97	KC578579|Kerivoula argentata|South Africa	91.82	KC578572|Eptesicus hottentotus|South Africa
05VZ_75_98_L10_R6_VN	77.78	KC578575|Nycteris thebaica|South Africa	90.72	KC578572|Eptesicus hottentotus|South Africa
05VZ_75_98_L15_R6_VN	80.93	KC578572|Eptesicus hottentotus|South Africa	94.64	KC578572|Eptesicus hottentotus|South Africa
05VZ_75_99_L01_R6_VN	80.68	KC578572|Eptesicus hottentotus|South Africa	92.75	KC578572|Eptesicus hottentotus|South Africa
05VZ_75_99_L02_R6_VN	80.84	KC578575|Nycteris thebaica|South Africa	94.06	KC578572|Eptesicus hottentotus|South Africa
05VZ_75_99_L05_R6_VN	81.97	KC578579|Kerivoula argentata|South Africa	92.86	KC578572|Eptesicus hottentotus|South Africa

**Figure 2:**
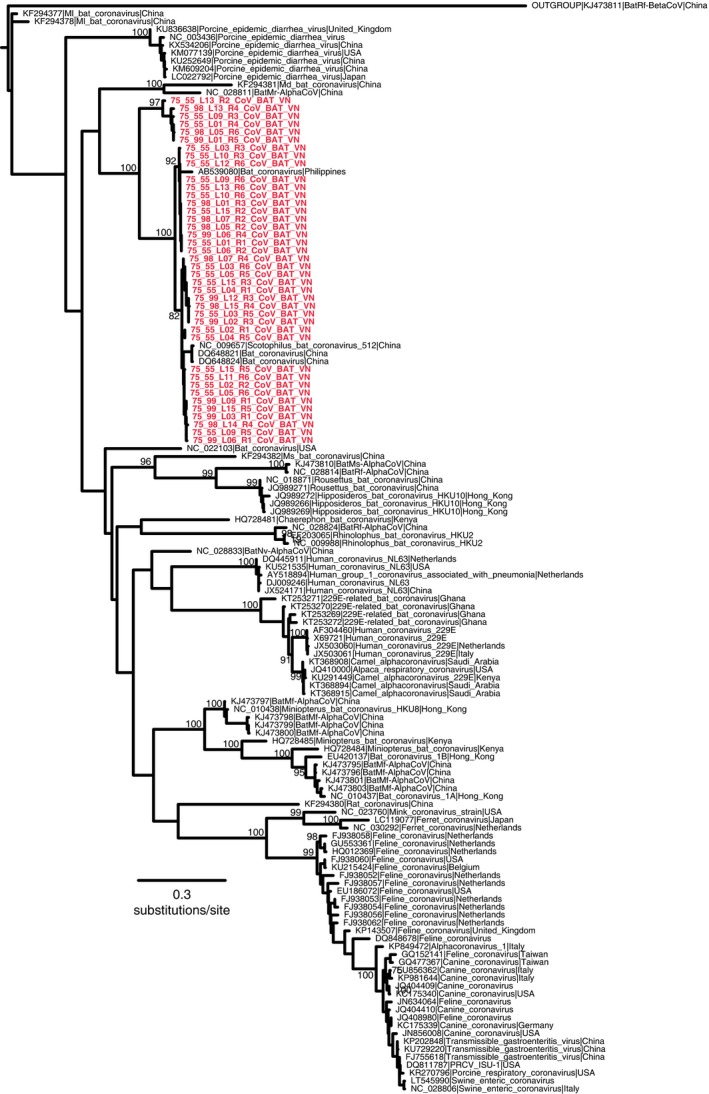
The phylogenetic relationships of alphacoronaviruses sampled from Vietnamese bats. Maximum‐likelihood phylogeny constructed using RNA sequences from 40 Vietnamese bats and 98 reference sequences from the conserved region within the coronavirus RNA‐dependent *RNA polymerase* (RdRp) gene (407 bp) accessed from GenBank, including a bat betacoronavirus sequence as an outgroup. Scale bar indicates the number of substitutions per site. Bootstrap support values are shown for nodes with ≥80% bootstrap support. Sequences from Vietnamese bats are indicated in red. [Colour figure can be viewed at http://wileyonlinelibrary.com]

**Figure 3:**
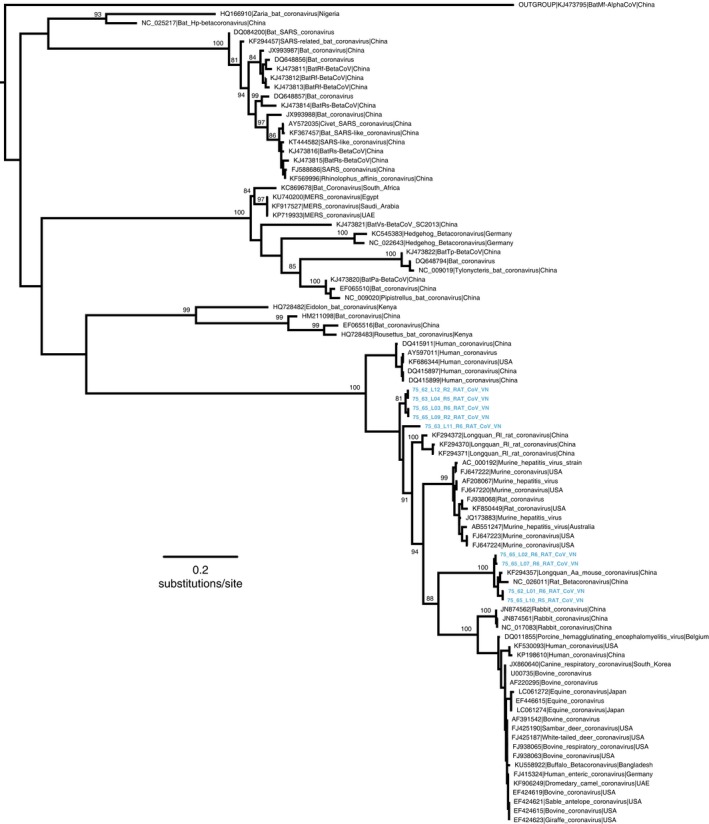
The phylogenetic relationships of betacoronaviruses sampled from Vietnamese rats. Maximum‐likelihood phylogeny constructed using RNA sequences from nine Vietnamese rats and 81 reference sequences from the conserved region within the coronavirus RNA‐dependent *RNA polymerase* (RdRp) gene (407 bp) accessed from GenBank, including a bat alphacoronavirus as an outgroup. Scale bar indicates the number of substitutions per site. Bootstrap support values are shown for nodes with ≥80% bootstrap support. Sequences from Vietnamese rats are indicated in blue. [Colour figure can be viewed at http://wileyonlinelibrary.com]

Sequence data indicated that all bat coronaviruses characterized in this study were of the alphacoronavirus lineage, while all rat coronaviruses were of the betacoronavirus lineage. The alphacoronavirus sequences obtained from bat samples (*n* = 55; Table 2 and S5) and betacoronavirus sequences from rat samples (*n* = 12; Table 3 and S6) (GenBank accession numbers: KX092163–KX092228) were aligned with reference sequences (407 bp), identical sequences and lower quality sequences were removed, and ML trees were constructed. Based on the 2009 ITCV classification, none of the coronaviruses detected in this study represented novel species (Tables 2 and 3). Phylogenetic reconstruction indicated that bat alphacoronaviruses from Vietnam clustered with other bat viruses detected in China and the Philippines (Figure 2a), and showed the highest amino acid identity to bat alphacoronaviruses described in China (Table 2). The betacoronaviruses detected in rats in this study clustered with reference samples originating from other mammal populations, with one of the two clusters found to be closely related to betacoronaviruses detected in rodent populations sampled in China (Figure 3). These sequences showed the highest identity to rat and mouse betacoronaviruses from China (Table 3). Again, inference is somewhat limited by the length of these sequences and additional sequencing effort is needed to confirm these preliminary findings.

**Table 2:** Maximum nucleotide and amino acid identities of Vietnamese bat alphacoronavirus sequences relative to other known alphacoronaviruses

Alphacoronavirus	Highest nucleotide identity (%)	Closest match	Highest amino acid identity (%)	Closest match
75_55_L01_R1_CoV_BAT_VN	95.55	AB539080|Scotophilus kuhlii|Philippines	97.78	NC_009657|Scotophilus spp.|China, DQ648821|Unknown bat species|China
75_55_L03_R3_CoV_BAT_VN	95.81	AB539080|Scotophilus kuhlii|Philippines	98.52	NC_009657|Scotophilus spp.|China, DQ648821|Unknown bat species|China
75_55_L06_R2_CoV_BAT_VN	95.81	AB539080|Scotophilus kuhlii|Philippines	98.52	NC_009657|Scotophilus spp.|China, DQ648821|Unknown bat species|China
75_55_L09_R6_CoV_BAT_VN	96.34	AB539080|Scotophilus kuhlii|Philippines	98.52	NC_009657|Scotophilus spp.|China, DQ648821|Unknown bat species|China
75_55_L10_R3_CoV_BAT_VN	96.07	AB539080|Scotophilus kuhlii|Philippines	98.52	NC_009657|Scotophilus spp.|China, DQ648821|Unknown bat species|China
75_55_L10_R6_CoV_BAT_VN	95.81	AB539080|Scotophilus kuhlii|Philippines	98.52	NC_009657|Scotophilus spp.|China, DQ648821|Unknown bat species|China
75_55_L12_R6_CoV_BAT_VN	96.07	AB539080|Scotophilus kuhlii|Philippines	97.78	NC_009657|Scotophilus spp.|China, DQ648821|Unknown bat species|China
75_55_L13_R6_CoV_BAT_VN	96.34	AB539080|Scotophilus kuhlii|Philippines	98.52	NC_009657|Scotophilus spp.|China, DQ648821|Unknown bat species|China
75_55_L15_R2_CoV_BAT_VN	96.07	AB539080|Scotophilus kuhlii|Philippines	98.52	NC_009657|Scotophilus spp.|China, DQ648821|Unknown bat species|China
75_98_L07_R5_CoV_BAT_VN	94.85	AB539080|Scotophilus kuhlii|Philippines	97.44	NC_009657|Scotophilus spp.|China, DQ648821|Unknown bat species|China
75_98_L01_R3_CoV_BAT_VN	95.81	AB539080|Scotophilus kuhlii|Philippines	98.52	NC_009657|Scotophilus spp.|China, DQ648821|Unknown bat species|China
75_98_L05_R2_CoV_BAT_VN	95.81	AB539080|Scotophilus kuhlii|Philippines	97.78	NC_009657|Scotophilus spp.|China, DQ648821|Unknown bat species|China
75_98_L07_R2_CoV_BAT_VN	96.07	AB539080|Scotophilus kuhlii|Philippines	98.52	NC_009657|Scotophilus spp.|China, DQ648821|Unknown bat species|China
75_99_L06_R4_CoV_BAT_VN	95.55	AB539080|Scotophilus kuhlii|Philippines	98.52	NC_009657|Scotophilus spp.|China, DQ648821|Unknown bat species|China
75_55_L01_R4_CoV_BAT_VN	84.44	DQ648821|Unknown bat species|China	93.33	NC_009657|Scotophilus spp.|China, DQ648821|Unknown bat species|China
75_55_L02_R1_CoV_BAT_VN	96.30	DQ648821|Unknown bat species|China	100	NC_009657|Scotophilus spp.|China, DQ648821|Unknown bat species|China
75_55_L02_R2_CoV_BAT_VN	97.04	DQ648821|Unknown bat species|China	100	NC_009657|Scotophilus spp.|China, DQ648821|Unknown bat species|China
75_55_L03_R5_CoV_BAT_VN	95.31	DQ648821|Unknown bat species|China	100	NC_009657|Scotophilus spp.|China, DQ648821|Unknown bat species|China
75_55_L03_R6_CoV_BAT_VN	95.80	DQ648821|Unknown bat species|China	100	NC_009657|Scotophilus spp.|China, DQ648821|Unknown bat species|China
75_55_L04_R1_CoV_BAT_VN	96.30	DQ648821|Unknown bat species|China	100	NC_009657|Scotophilus spp.|China, DQ648821|Unknown bat species|China
75_55_L04_R5_CoV_BAT_VN	96.30	DQ648821|Unknown bat species|China	100	NC_009657|Scotophilus spp.|China, DQ648821|Unknown bat species|China
75_55_L05_R5_CoV_BAT_VN	95.56	DQ648821|Unknown bat species|China	99.26	NC_009657|Scotophilus spp.|China, DQ648821|Unknown bat species|China
75_55_L05_R6_CoV_BAT_VN	97.04	DQ648821|Unknown bat species|China	100	NC_009657|Scotophilus spp.|China, DQ648821|Unknown bat species|China
75_55_L09_R3_CoV_BAT_VN	84.69	DQ648821|Unknown bat species|China	93.33	NC_009657|Scotophilus spp.|China, DQ648821|Unknown bat species|China
75_55_L09_R5_CoV_BAT_VN	96.79	DQ648821|Unknown bat species|China	99.26	NC_009657|Scotophilus spp.|China, DQ648821|Unknown bat species|China
75_55_L11_R6_CoV_BAT_VN	96.78	DQ648821|Unknown bat species|China	100	NC_009657|Scotophilus spp.|China, DQ648821|Unknown bat species|China
75_55_L13_R2_CoV_BAT_VN	85.68	DQ648821|Unknown bat species|China	94.82	NC_009657|Scotophilus spp.|China, DQ648821|Unknown bat species|China
75_55_L13_R4_CoV_BAT_VN	97.04	DQ648821|Unknown bat species|China	100	NC_009657|Scotophilus spp.|China, DQ648821|Unknown bat species|China
75_55_L14_R4_CoV_BAT_VN	96.34	DQ648821|Unknown bat species|China	100	NC_009657|Scotophilus spp.|China, DQ648821|Unknown bat species|China
75_55_L15_R3_CoV_BAT_VN	96.30	DQ648821|Unknown bat species|China	100	NC_009657|Scotophilus spp.|China, DQ648821|Unknown bat species|China
75_55_L15_R5_CoV_BAT_VN	96.79	DQ648821|Unknown bat species|China	100	NC_009657|Scotophilus spp.|China, DQ648821|Unknown bat species|China
75_98_L05_R6_CoV_BAT_VN	84.44	DQ648821|Unknown bat species|China	94.07	NC_009657|Scotophilus spp.|China, DQ648821|Unknown bat species|China
75_98_L07_R4_CoV_BAT_VN	96.30	DQ648821|Unknown bat species|China	100	NC_009657|Scotophilus spp.|China, DQ648821|Unknown bat species|China
75_98_L10_R4_CoV_BAT_VN	97.04	DQ648821|Unknown bat species|China	100	NC_009657|Scotophilus spp.|China, DQ648821|Unknown bat species|China
75_98_L13_R4_CoV_BAT_VN	84.20	DQ648821|Unknown bat species|China	91.85	NC_009657|Scotophilus spp.|China, DQ648821|Unknown bat species|China
75_98_L14_R2_CoV_BAT_VN	97.04	DQ648821|Unknown bat species|China	100.00	NC_009657|Scotophilus spp.|China, DQ648821|Unknown bat species|China
75_98_L14_R4_CoV_BAT_VN	96.54	DQ648821|Unknown bat species|China	97.78	NC_009657|Scotophilus spp.|China, DQ648821|Unknown bat species|China
75_98_L15_R4_CoV_BAT_VN	95.31	DQ648821|Unknown bat species|China	100	NC_009657|Scotophilus spp.|China, DQ648821|Unknown bat species|China
75_98_L15_R5_CoV_BAT_VN	96.40	DQ648821|Unknown bat species|China	100	NC_009657|Scotophilus spp.|China, DQ648821|Unknown bat species|China
75_99_L02_R3_CoV_BAT_VN	95.56	DQ648821|Unknown bat species|China	100	NC_009657|Scotophilus spp.|China, DQ648821|Unknown bat species|China
75_99_L03_R1_CoV_BAT_VN	96.79	DQ648821|Unknown bat species|China	99.26	NC_009657|Scotophilus spp.|China, DQ648821|Unknown bat species|China
75_99_L06_R1_CoV_BAT_VN	97.04	DQ648821|Unknown bat species|China	99.26	NC_009657|Scotophilus spp.|China, DQ648821|Unknown bat species|China
75_99_L07_R5_CoV_BAT_VN	96.66	DQ648821|Unknown bat species|China	100	NC_009657|Scotophilus spp.|China, DQ648821|Unknown bat species|China
75_99_L09_R1_CoV_BAT_VN	96.30	DQ648821|Unknown bat species|China	99.26	NC_009657|Scotophilus spp.|China, DQ648821|Unknown bat species|China
75_99_L12_R3_CoV_BAT_VN	95.56	DQ648821|Unknown bat species|China	100	NC_009657|Scotophilus spp.|China, DQ648821|Unknown bat species|China
75_99_L14_R1_CoV_BAT_VN	96.30	DQ648821|Unknown bat species|China	100	NC_009657|Scotophilus spp.|China, DQ648821|Unknown bat species|China
75_99_L15_R3_CoV_BAT_VN	95.56	DQ648821|Unknown bat species|China	100	NC_009657|Scotophilus spp.|China, DQ648821|Unknown bat species|China
75_99_L15_R5_CoV_BAT_VN	96.27	DQ648821|Unknown bat species|China	99.25	NC_009657|Scotophilus spp.|China, DQ648821|Unknown bat species|China
75_99_L01_R5_CoV_BAT_VN	84.44	DQ648821|Unknown bat species|China	94.07	NC_009657|Scotophilus spp.|China, DQ648821|Unknown bat species|China

**Table 3:** Maximum nucleotide and amino acid identities of Vietnamese rat betacoronavirus sequences relative to other known betacoronaviruses

Betacoronavirus	Highest nucleotide identity (%)	Closest match	Highest amino acid identity (%)	Closest match
75_62_L01_R6_RAT_CoV_VN	97.54	KF294357|Apodemus agrarius|China	100	KF294357|Apodemus agrarius|China, NC_026011|Rattus norvegicus|China
75_62_L12_R2_RAT_CoV_VN	93.86	KF294372|Niviventer confucianus|China	98.52	KF294370|Rattus tanezumi|China, KF294372|Niviventer confucianus|China
75_63_L04_R5_RAT_CoV_VN	93.86	KF294372|Niviventer confucianus|China	98.52	KF294370|Rattus tanezumi|China, KF294372|Niviventer confucianus|China
75_63_L11_R6_RAT_CoV_VN	90.66	KF294372|Niviventer confucianus|China	94.82	KF294370|Rattus tanezumi|China, KF294372|Niviventer confucianus|China
75_65_L02_R6_RAT_CoV_VN	97.54	KF294357|Apodemus agrarius|China	99.26	KF294357|Apodemus agrarius|China, NC_026011|Rattus norvegicus|China
75_65_L03_R6_RAT_CoV_VN	93.37	KF294372|Niviventer confucianus|China	97.04	KF294370|Rattus tanezumi|China, KF294372|Niviventer confucianus|China
75_65_L07_R5_RAT_CoV_VN	97.79	KF294357|Apodemus agrarius|China	99.04	KF294357|Apodemus agrarius|China, NC_026011|Rattus norvegicus|China
75_65_L07_R6_RAT_CoV_VN	97.30	KF294357|Apodemus agrarius|China	98.52	KF294357|Apodemus agrarius|China, NC_026011|Rattus norvegicus|China
75_65_L09_R2_RAT_CoV_VN	93.12	KF294372|Niviventer confucianus|China	96.30	KF294370|Rattus tanezumi|China, KF294372|Niviventer confucianus|China
75_65_L10_R5_RAT_CoV_VN	97.79	KF294357|Apodemus agrarius|China	100	KF294357|Apodemus agrarius|China, NC_026011|Rattus norvegicus|China

## 4 Discussion

Here, we sought to investigate the prevalence of paramyxovirus, coronavirus and filovirus in bat and rat populations in southern Viet Nam. For this study, we implemented a sampling and testing protocol that sought to maximize the likelihood of detection if the target viruses were present. Firstly, we collected pooled urine and faecal samples from three different bat guano farms in the south of Viet Nam, rather than catching and sampling individual animals, so each sample potentially represented multiple animals. Bats forage nocturnally and roost communally in trees during the day; during this period, they frequently urinate and defecate, allowing collection on plastic sheeting. This approach has been used successfully (Field et al., 2015) and has been shown to increase the number of animals sampled and therefore increase the chance of detecting viral nucleic acid. A limitation of this investigation is that the pooling of samples from many animals may have overestimated the true prevalence, given the possibility that multiple positive (pooled) samples may reflect a single individual.

The methods used in the current study resulted in the identification of previously unidentified paramyxoviruses and coronaviruses in Viet Nam and has expanded our knowledge of circulation of viruses with zoonotic potential in the bat and rat population in Viet Nam. Recently, bats have been shown to harbour a great diversity of previously unknown paramyxoviruses, some of which have been associated with zoonotic events (Baker et al., 2013). The interrelationship of these paramyxoviruses with bats highlights the complex role that bats play as a reservoir for these viruses. Despite our sampling framework being narrow, it was relatively thorough, as we screened 248 bat faecal samples and 222 bat urine samples. RNA from novel paramyxoviruses was detected by PCR and could be characterized by sequencing in approximately 2% of bat faecal samples and nearly 5% of bat urine samples. Our study and studies by others indicate that it is highly likely that there are more extant bat‐derived paramyxoviruses yet to be characterized (Drexler et al., 2009, 2012; Lau et al., 2010). For this reason, it is crucial that the potential consequences and risk of zoonotic spillover of such viruses are investigated further.

Our data additionally suggested that alphacoronaviruses are constantly circulating in the bat population in Vietnam. The presence of coronavirus RNA in 22% of bat samples showed that coronaviruses may be endemic, with highly similar viruses circulating on all of the sampled farms at different time points. Ge et al., (2016) recently observed similar characteristics for coronaviruses in Chinese bats. Phylogenetic analysis revealed that the sequences generated here belong to a recently detected and unclassified bat betacoronavirus lineage, and are closely related to other bat coronaviruses sampled in China and the Philippines (Tang et al., 2006; Watanabe et al., 2010).

Using our sampling framework, we found that our ability to detect paramyxoviruses and coronaviruses was not associated with location or time of sample collection, and appeared to be both random and sporadic. Our findings indicate that the novel paramyxoviruses detected were not present in all bat colonies at the time of sampling, suggesting that the extent of carriage and/or excretion in any particular bat colony is likely to fluctuate over time. These data indicated that while infection in a bat colony is periodic and transient for paramyxoviruses, this may not be the case for coronaviruses. Therefore, from a zoonotic infection risk management perspective, it is prudent to assume that any bat colony could be infected at any time and that those working in proximity to the bat colonies should adopt exposure risk minimization strategies in case these viruses are capable of infecting humans. We additionally found that PCR amplification‐positive samples from bats were associated with sample type; urine was more likely to be positive for paramyxoviruses while faecal material was more likely to generate positive amplification of coronaviruses. Importantly, the screening of these samples by PCR alone may underestimate the prevalence of these viruses in the sampled populations, as detection is limited by the specificity of primers and the quality of samples collected using indirect methods such as that used for the collection of bat samples.

Although none of the bat samples tested in this study indicate the presence of filoviruses, this does not provide conclusive evidence that these viruses are not circulating in Viet Nam. While fruit bats have been implicated as the primary reservoirs of filoviruses, a previous survey of multiple bat species in the Philippines confirmed Ebola Reston virus infection in one insectivorous bat species (*Miniopterus schreibersii*) of 21 insectivorous and fruit‐eating bat species sampled (Jayme et al., 2015; Leendertz, Gogarten, Düx, Calvignac‐Spencer, & Leendertz, 2015). The samples included in the current study were collected only from the insectivorous *S. kuhlii*, while at least 90 other chiropterans have also been identified in Viet Nam (http://www.iucnredlist.org/). Our sampling was further limited by the use of faecal and urine samples, which are unlikely to be optimum materials in which to detect filoviruses. Thus, a much larger and more comprehensive sampling of multiple bat species using more direct sampling methods would be necessary to detect and understand the potential circulation of filoviruses in the region.

In contrast to previous investigations (Woo et al., 2012), paramyxovirus nucleic acid was not detected in any the rat faecal samples tested. A possible explanation as to why the rats tested were all negative for paramyxoviruses could be that the sample type (faeces) is not appropriate to detect the virus. This observation is supported by a previous study, where a novel rat paramyxovirus was detected only in the kidney and spleen of rats (Woo et al., 2012). Novel coronavirus RNA was detected by PCR amplification and characterized by sequencing in 4.4% of the rat faeces. The obtained rat coronavirus sequences were closely related to rat and murine betacoronavirus sequences from southern China that were found to be capable of infecting up to ten different rat species, including *R. losea* and *R. tanezumi* (Wang et al., 2015). In our study, novel rat coronavirus strains were detected in a single rat species (*R. argentiventer*). These data do not exclude the possibility that other rat species carry this coronavirus virus, particularly as our sampling was biased towards *R. argentiventer* (234/270).

Despite a lack of appropriate control measures for zoonotic viruses in Viet Nam, viral spillover from bats and/or rats has not, as yet, been observed, although these preliminary findings suggest that both coronaviruses and paramyxoviruses may be endemic in Vietnamese bat populations. While we are unable to comment on the pathogenic potential of these novel viruses in humans, it is likely that if such infections do arise, they are rare and go undiagnosed. Paramyxoviruses in particular are known to cause infections in humans, and the spillover of Hendra virus and Nipah virus from bats to horses or pigs, respectively, and subsequently to humans has been reported in Australia and Malaysia (Edson et al., 2015; Goldspink et al., 2015; Sherrini & Chong, 2014). Laboratory investigations are being conducted to assess whether the coronaviruses and paramyxoviruses detected here are viable in cell culture in order to better predict whether these viruses have the potential to cause spillover infections in the human population and to improve genomic characterization of these viruses to allow for greater phylogenetic inference. Furthermore, our countrywide project is aiming to recruit hospitalized patients with common and uncommon disease phenotypes in order to determine whether such viruses cause previously undiagnosed infections in humans in Viet Nam (Rabaa et al., 2015).

In conclusion, we have detected the presence of bat paramyxoviruses, bat coronaviruses and rodent‐associated coronaviruses within a limited geographic area and time frame in Viet Nam. Consequently, we suggest that larger‐scale surveillance is needed to fully understand the role played by rodents and bats in the evolution, emergence and dissemination of paramyxoviruses and coronaviruses in Viet Nam.

## Competing interests

The authors state that they have no competing interests.

## Appendix 1

### The VIZIONS Consortium members are

**Oxford University Clinical Research Unit** (alphabetic order by surname): Bach Tuan Kiet, Stephen Baker, Alessandra Berto, Maciej F. Boni, Juliet E Bryant, Bui Duc Phu, James I Campbell, Juan Carrique‐Mas, Dang Manh Hung, Dang Thao Huong, Dang Tram Oanh, Jeremy N. Day, Dinh Van Tan, H. Rogier van Doorn, Duong An Han, Jeremy J Farrar, Hau Thi Thu Trang, Ho Dang Trung Nghia, Hoang Bao Long, Hoang Van Duong, Huynh Thi Kim Thu, Lam Chi Cuong, Le Manh Hung, Le Thanh Phuong, Le Thi Phuc, Le Thi Phuong, Le Xuan Luat, Luu Thi Thu Ha, Ly Van Chuong, Mai Thi Phuoc Loan, Behzad Nadjm, Ngo Thanh Bao, Ngo Thi Hoa, Ngo Tri Tue, Nguyen Canh Tu, Nguyen Dac Thuan, Nguyen Dong, Nguyen Khac Chuyen, Nguyen Ngoc An, Nguyen Ngoc Vinh, Nguyen Quoc Hung, Nguyen Thanh Dung, Nguyen Thanh Minh, Nguyen Thi Binh, Nguyen Thi Hong Tham, Nguyen Thi Hong Tien, Nguyen Thi Kim Chuc, Nguyen Thi Le Ngoc, Nguyen Thi Lien Ha, Nguyen Thi Nam Lien, Nguyen Thi Ngoc Diep, Nguyen Thi Nhung, Nguyen Thi Song Chau, Nguyen Thi Yen Chi, Nguyen Thieu Trinh, Nguyen Thu Van, Nguyen Van Cuong, Nguyen Van Hung, Nguyen Van Kinh, Nguyen Van Minh Hoang, Nguyen Van My, Nguyen Van Thang, Nguyen Van Thanh, Nguyen Van Vinh Chau, Nguyen Van Xang, Pham Ha My, Pham Hong Anh, Pham Thi Minh Khoa, Pham Thi Thanh Tam, Pham Van Lao, Pham Van Minh, Phan Van Be Bay, Maia A Rabaa, Motiur Rahman, Corinne Thompson, Guy Thwaites, Ta Thi Dieu Ngan, Tran Do Hoang Nhu, Tran Hoang Minh Chau, Tran Khanh Toan, Tran My Phuc, Tran Thi Kim Hong, Tran Thi Ngoc Dung, Tran Thi Thanh Thanh, Tran Thi Thuy Minh, Tran Thua Nguyen, Tran Tinh Hien, Trinh Quang Tri, Vo Be Hien, Vo Nhut Tai, Vo Quoc Cuong, Voong Vinh Phat, Vu Thi Lan Huong, Vu Thi Ty Hang, Heiman Wertheim. **Centre for Immunity, Infection and Evolution, University of Edinburgh** (alphabetic order by surname): Carlijn Bogaardt, Liam Brierley, Margo Chase‐Topping, Al Ivens, Lu Lu, Dung Nguyen, Andrew Rambaut, Peter Simmonds, Mark Woolhouse. **The Wellcome Trust Sanger Institute, Hinxton, United Kingdom** (alphabetic order by surname): Matthew Cotten, Bas B. Oude Munnink, Paul Kellam, My Vu Tra Phan. **Laboratory of Experimental Virology, Department of Medical Microbiology, Center for Infection and Immunity Amsterdam (CINIMA), Academic Medical Center of the University of Amsterdam, Amsterdam, the Netherlands** (alphabetic order by surname): Lia van der Hoek, Martin Deijs, Maarten F. Jebbink, Seyed Mohammad Jazaeri Farsani. **Metabiota, California, United States of America** (alphabetic order by surname): Karen Saylors, Nathan Wolfe.
