# Supplementary material for: Detection of potentially novel paramyxovirus and coronavirus viral RNA in bats and rats in the Mekong Delta region of southern Viet Nam
Source: Zoonoses Public Health. 2017 Apr 18;65(1):30–42. doi: 10.1111/zph.12362 (PMC5811810; doi:10.1111/zph.12362)
Supplement: Supplementary file 1 [file ZPH-65-30-s001.docx]

**Supplementary Tables**

**Supplementary Table S1.** Coronavirus and paramyxovirus detection in bat fecal samples according to site and sampling round

| **Tested viruses** | | **Round 1** | | **Round 2** | | **Round 3** | | **Round 4** | | **Round 5** | | **Round 6** | |
| --- | --- | --- | --- | --- | --- | --- | --- | --- | --- | --- | --- | --- | --- |
|  |  | Negative | Positive | Negative | Positive | Negative | Positive | Negative | Positive | Negative | Positive | Negative | Positive |
| Coronavirus | Farm 1 | 5 (50%) | 5 (50%) | 10 (66.67%) | 5 (33.33%) | 11 (73.33%) | 4 (26.67%) | 11 (73.33%) | 4 (26.67%) | 8 (57.14%) | 6 (42.86%) | 7 (46.67%) | 8 (53.33%) |
|  | Farm 2 | 0 (0%) | 0 (0%) | 12 (80%) | 3 (20%) | 14 (93.33%) | 1 (6.67%) | 9 (60%) | 6 (40%) | 13 (86.67%) | 2 (13.33%) | 13 (86.67%) | 2 (13.33%) |
|  | Farm 3 | 11 (73.33%) | 4 (26.67%) | 15 (100%) | 0 (0%) | 12 (80%) | 3 (20%) | 14 (93.33%) | 1 (6.67%) | 12 (80%) | 3 (20%) | 13 (86.67%) | 2 (13.33%) |
| Paramyxovirus | Farm 1 | 10 (100%) | 0 (0%) | 14 (93.33%) | 1 (6.67%) | 13 (86.67%) | 2 (13.33%) | 15 (100%) | 0 (0%) | 15 (100%) | 0 (0%) | 14 (93.33%) | 1 (6.67%) |
|  | Farm 2 | 0 (0%) | 0 (0%) | 12 (80%) | 3 (20%) | 14 (93.33%) | 1 (6.67%) | 15 (100%) | 0 (0%) | 15 (100%) | 0 (0%) | 14 (93.33%) | 1 (6.67%) |
|  | Farm 3 | 15 (100%) | 0 (0%) | 15 (100%) | 0 (0%) | 15 (100%) | 0 (0%) | 15 (100%) | 0 (0%) | 15 (100%) | 0 (0%) | 14 (93.33%) | 1 (6.67%) |

**Supplementary Table S2.** Coronavirus and paramyxovirus detection in bat urine samples according to site and sampling round

| **Tested viruses** | | **Round 2** | | **Round 3** | | **Round 4** | | **Round 5** | | **Round 6** | |
| --- | --- | --- | --- | --- | --- | --- | --- | --- | --- | --- | --- |
|  |  | Negative | Positive | Negative | Positive | Negative | Positive | Negative | Positive | Negative | Positive |
| Coronavirus | Farm 1 | 15 (100%) | 0 (0%) | 15 (100%) | 0 (0%) | 12 (80%) | 3 (20%) | 11 (78.57%) | 3 (21.43%) | 15 (100%) | 0 (0%) |
|  | Farm 2 | 15 (100%) | 0 (0%) | 14 (93.33%) | 1 (6.67%) | 13 (86.67%) | 2 (13.33%) | 13 (100%) | 0 (0%) | 15 (100%) | 0 (0%) |
|  | Farm 3 | 15 (100%) | 0 (0%) | 14 (93.33%) | 1 (6.67%) | 11 (73.33%) | 4 (26.67%) | 14 (93.33%) | 1 (6.67%) | 15 (100%) | 0 (0%) |
| Paramyxovirus | Farm 1 | 15 (100%) | 0 (0%) | 15 (100%) | 0 (0%) | 15 (100%) | 0 (0%) | 13 (92.86%) | 1 (7.14%) | 11 (73.33%) | 4 (26.67%) |
|  | Farm 2 | 14 (93.33%) | 1 (6.67%) | 15 (100%) | 0 (0%) | 15 (100%) | 0 (0%) | 12 (92.31%) | 1 (7.69%) | 9 (60%) | 6 (40%) |
|  | Farm 3 | 15 (100%) | 0 (0%) | 15 (100%) | 0 (0%) | 15 (100%) | 0 (0%) | 14 (93.33%) | 1 (6.67%) | 9 (60%) | 6 (40%) |

**Supplementary Table S3.** Coronavirus and paramyxovirus detection in rat fecal samples according to site and sampling round

| **Tested viruses** | | **Round 1** | | **Round 2** | | **Round 3** | | **Round 4** | | **Round 5** | | **Round 6** | |
| --- | --- | --- | --- | --- | --- | --- | --- | --- | --- | --- | --- | --- | --- |
|  |  | Negative | Positive | Negative | Positive | Negative | Positive | Negative | Positive | Negative | Positive | Negative | Positive |
| Coronavirus | Market 1 | 14 (93.33%) | 1 (6.67%) | 7 (46.67%) | 8 (53.33%) | 15 (100%) | 0 (0%) | 15 (100%) | 0 (0%) | 15 (100%) | 0 (0%) | 13 (86.67%) | 2 (13.33%) |
|  | Market 2 | 15 (100%) | 0 (0%) | 14 (93.33%) | 1 (6.67%) | 15 (100%) | 0 (0%) | 14 (93.33%) | 1 (6.67%) | 12 (80%) | 3 (20%) | 14 (93.33%) | 1 (6.67%) |
|  | Market 3 | 15 (100%) | 0 (0%) | 14 (93.33%) | 1 (6.67%) | 12 (80%) | 3 (20%) | 15 (100%) | 0 (0%) | 13 (86.67%) | 2 (13.33%) | 12 (80%) | 3 (20%) |
| Paramyxovirus | Market 1 | 15 (100%) | 0 (0%) | 15 (100%) | 0 (0%) | 15 (100%) | 0 (0%) | 15 (100%) | 0 (0%) | 14 (93.33%) | 1 (6.67%) | 15 (100%) | 0 (0%) |
|  | Market 2 | 15 (100%) | 0 (0%) | 15 (100%) | 0 (0%) | 15 (100%) | 0 (0%) | 15 (100%) | 0 (0%) | 14 (93.33%) | 1 (6.67%) | 15 (100%) | 0 (0%) |
|  | Market 3 | 15 (100%) | 0 (0%) | 15 (100%) | 0 (0%) | 15 (100%) | 0 (0%) | 15 (100%) | 0 (0%) | 15 (100%) | 0 (0%) | 15 (100%) | 0 (0%) |
|  |  |  |  |  |  |  |  |  |  |  |  |  |  |

**Supplementary Table S4. Bat paramyxovirus sequences generated in this study**

| Sample | Country | Year | Bat species | Accession number |
| --- | --- | --- | --- | --- |
| 7598L05R2PARABATVN | Vietnam | 2012-2014 | *Scotophilus kuhlii* | KX092148 |
| 7598L06R2PaRABATVN | Vietnam | 2012-2014 | *Scotophilus kuhlii* | KX092149 |
| 7555L09R3PARABATVN | Vietnam | 2012-2014 | *Scotophilus kuhlii* | KX092150 |
| 7598L02R3PARABATVN | Vietnam | 2012-2014 | *Scotophilus kuhlii* | KX092151 |
| 05VZ7599L13R6 | Vietnam | 2012-2014 | *Scotophilus kuhlii* | KX092152 |
| 05VZ7555L10R6 | Vietnam | 2012-2014 | *Scotophilus kuhlii* | KX092153 |
| 05VZ7555L13R6 | Vietnam | 2012-2014 | *Scotophilus kuhlii* | KX092154 |
| 05VZ7598L05R6 | Vietnam | 2012-2014 | *Scotophilus kuhlii* | KX092155 |
| 05VZ7598L08R6 | Vietnam | 2012-2014 | *Scotophilus kuhlii* | KX092156 |
| 05VZ7598L09 | Vietnam | 2012-2014 | *Scotophilus kuhlii* | KX092157 |
| 05VZ7598L10R6 | Vietnam | 2012-2014 | *Scotophilus kuhlii* | KX092158 |
| 05VZ7598L15R6F | Vietnam | 2012-2014 | *Scotophilus kuhlii* | KX092159 |

**Supplementary Table S5. Bat coronavirus sequences generated in this study**

| Sample | Country | Year | Bat species | Accession number |
| --- | --- | --- | --- | --- |
| 75-55-L01-R1-CoV-BAT-VN | Vietnam | 2012-2014 | *Scotophilus kuhlii* | KX092163 |
| 75-55-L04-R1-CoV-BAT-VN | Vietnam | 2012-2014 | *Scotophilus kuhlii* | KX092164 |
| 75-55-L07-R1-CoV-BAT-VN | Vietnam | 2012-2014 | *Scotophilus kuhlii* | KX092165 |
| 75-99-L03-R1-CoV-BAT-VN | Vietnam | 2012-2014 | *Scotophilus kuhlii* | KX092166 |
| 75-99-L06-R1-CoV-BAT-VN | Vietnam | 2012-2014 | *Scotophilus kuhlii* | KX092167 |
| 75-55-L10-R1-CoV-BAT-VN | Vietnam | 2012-2014 | *Scotophilus kuhlii* | KX092168 |
| 75-99-L09-R1-CoV-BAT-VN | Vietnam | 2012-2014 | *Scotophilus kuhlii* | KX092169 |
| 75-99-L14-R1-CoV-BAT-VN | Vietnam | 2012-2014 | *Scotophilus kuhlii* | KX092170 |
| 75-55-L02-R2-CoV-BAT-VN | Vietnam | 2012-2014 | *Scotophilus kuhlii* | KX092171 |
| 75-55-L06-R2-CoV-BAT-VN | Vietnam | 2012-2014 | *Scotophilus kuhlii* | KX092172 |
| 75-55-L08-R2-CoV-BAT-VN | Vietnam | 2012-2014 | *Scotophilus kuhlii* | KX092173 |
| 75-55-L13-R2-CoV-BAT-VN | Vietnam | 2012-2014 | *Scotophilus kuhlii* | KX092174 |
| 75-98-L05-R2-CoV-BAT-VN | Vietnam | 2012-2014 | *Scotophilus kuhlii* | KX092175 |
| 75-55-L15-R2-CoV-BAT-VN | Vietnam | 2012-2014 | *Scotophilus kuhlii* | KX092176 |
| 75-98-L07-R2-CoV-BAT-VN | Vietnam | 2012-2014 | *Scotophilus kuhlii* | KX092177 |
| 75-98-L14-R2-CoV-BAT-VN | Vietnam | 2012-2014 | *Scotophilus kuhlii* | KX092178 |
| 75-55-L03-R3-CoV-BAT-VN | Vietnam | 2012-2014 | *Scotophilus kuhlii* | KX092179 |
| 75-55-L09-R3-CoV-BAT-VN | Vietnam | 2012-2014 | *Scotophilus kuhlii* | KX092180 |
| 75-55-L10-R3-CoV-BAT-VN | Vietnam | 2012-2014 | *Scotophilus kuhlii* | KX092181 |
| 75-99-L12-R3-CoV-BAT-VN | Vietnam | 2012-2014 | *Scotophilus kuhlii* | KX092182 |
| 75-99-L15-R3-CoV-BAT-VN | Vietnam | 2012-2014 | *Scotophilus kuhlii* | KX092183 |
| 75-99-L02-R3-CoV-BAT-VN | Vietnam | 2012-2014 | *Scotophilus kuhlii* | KX092184 |
| 75-55-L15-R3-CoV-BAT-VN | Vietnam | 2012-2014 | *Scotophilus kuhlii* | KX092185 |
| 75-98-L01-R3-CoV-BAT-VN | Vietnam | 2012-2014 | *Scotophilus kuhlii* | KX092186 |
| 75-55-L13-R4-CoV-BAT-VN | Vietnam | 2012-2014 | *Scotophilus kuhlii* | KX092187 |
| 75-55-L14-R4-CoV-BAT-VN | Vietnam | 2012-2014 | *Scotophilus kuhlii* | KX092188 |
| 75-55-L01-R4-CoV-BAT-VN | Vietnam | 2012-2014 | *Scotophilus kuhlii* | KX092189 |
| 75-98-L07-R4-CoV-BAT-VN | Vietnam | 2012-2014 | *Scotophilus kuhlii* | KX092190 |
| 75-55-L03-R4-CoV-BAT-VN | Vietnam | 2012-2014 | *Scotophilus kuhlii* | KX092191 |
| 75-98-L10-R4-CoV-BAT-VN | Vietnam | 2012-2014 | *Scotophilus kuhlii* | KX092192 |
| 75-98-L12-R4-CoV-BAT-VN | Vietnam | 2012-2014 | *Scotophilus kuhlii* | KX092193 |
| 75-98-L13-R4-CoV-BAT-VN | Vietnam | 2012-2014 | *Scotophilus kuhlii* | KX092194 |
| 75-98-L14-R4-CoV-BAT-VN | Vietnam | 2012-2014 | *Scotophilus kuhlii* | KX092195 |
| 75-98-L15-R4-CoV-BAT-VN | Vietnam | 2012-2014 | *Scotophilus kuhlii* | KX092196 |
| 75-99-L06-R4-CoV-BAT-VN | Vietnam | 2012-2014 | *Scotophilus kuhlii* | KX092197 |
| 75-55-L04-R5-CoV-BAT-VN | Vietnam | 2012-2014 | *Scotophilus kuhlii* | KX092198 |
| 75-55-L03-R5-CoV-BAT-VN | Vietnam | 2012-2014 | *Scotophilus kuhlii* | KX092199 |
| 75-55-L05-R5-CoV-BAT-VN | Vietnam | 2012-2014 | *Scotophilus kuhlii* | KX092200 |
| 75-55-L09-R5-CoV-BAT-VN | Vietnam | 2012-2014 | *Scotophilus kuhlii* | KX092201 |
| 75-98-L15-R5-CoV-BAT-VN | Vietnam | 2012-2014 | *Scotophilus kuhlii* | KX092202 |
| 75-99-L07-R5-CoV-BAT-VN | Vietnam | 2012-2014 | *Scotophilus kuhlii* | KX092203 |
| 75-99-L15-R5-CoV-BAT-VN | Vietnam | 2012-2014 | *Scotophilus kuhlii* | KX092204 |
| 75-990L01-R5-CoV-BAT-VN | Vietnam | 2012-2014 | *Scotophilus kuhlii* | KX092205 |
| 75-98-L07-R5-CoV-BAT-VN | Vietnam | 2012-2014 | *Scotophilus kuhlii* | KX092206 |
| 75-55-L15-R5-CoV-BAT-VN | Vietnam | 2012-2014 | *Scotophilus kuhlii* | KX092207 |
| 75-55-L11-R6-CoV-BAT-VN | Vietnam | 2012-2014 | *Scotophilus kuhlii* | KX092208 |
| 75-55-L05-R6-CoV-BAT-VN | Vietnam | 2012-2014 | *Scotophilus kuhlii* | KX092209 |
| 75-55-L10-R6-CoV-BAT-VN | Vietnam | 2012-2014 | *Scotophilus kuhlii* | KX092210 |
| 75-55-L12-R6-CoV-BAT-VN | Vietnam | 2012-2014 | *Scotophilus kuhlii* | KX092211 |
| 75-98-L11-R6-CoV-BAT-VN | Vietnam | 2012-2014 | *Scotophilus kuhlii* | KX092212 |
| 75-55-L09-R6-CoV-BAT-VN | Vietnam | 2012-2014 | *Scotophilus kuhlii* | KX092213 |
| 75-55-L13-R6-CoV-BAT-VN | Vietnam | 2012-2014 | *Scotophilus kuhlii* | KX092214 |
| 75-55-L03-R6-CoV-BAT-VN | Vietnam | 2012-2014 | *Scotophilus kuhlii* | KX092215 |
| 75-98-L05-R6-CoV-BAT-VN | Vietnam | 2012-2014 | *Scotophilus kuhlii* | KX092216 |

**Supplementary Table S6. Rat coronavirus sequences generated in this study**

| Sample | Country | Year | Rat species | Accession number |
| --- | --- | --- | --- | --- |
| 7562L01R6RATCoV | Vietnam | 2012-2014 | *Rattus argentiventer* | KX092217 |
| 7562L01R2RATCoV | Vietnam | 2012-2014 | *Rattus argentiventer* | KX092218 |
| 7562L12R2RATCoV | Vietnam | 2012-2014 | *Rattus argentiventer* | KX092219 |
| 7563L04R5RATCoV | Vietnam | 2012-2014 | *Rattus argentiventer* | KX092220 |
| 7565L02R6RATCoV | Vietnam | 2012-2014 | *Rattus argentiventer* | KX092221 |
| 7565L03R6RATCoV | Vietnam | 2012-2014 | *Rattus argentiventer* | KX092222 |
| 7565L07R5RATCoV | Vietnam | 2012-2014 | *Rattus argentiventer* | KX092223 |
| 7565L09R2RATCoV | Vietnam | 2012-2014 | *Rattus argentiventer* | KX092224 |
| 7565L10R5RATCoV | Vietnam | 2012-2014 | *Rattus argentiventer* | KX092225 |
| 7563L11R5RATCoV | Vietnam | 2012-2014 | *Rattus argentiventer* | KX092226 |
| 7565L07R6RATCoV | Vietnam | 2012-2014 | *Rattus argentiventer* | KX092227 |
| 7563L11R6RATCoV | Vietnam | 2012-2014 | *Rattus argentiventer* | KX092228 |
